# A unique sarcopenic progression in the mouse rotator cuff

**DOI:** 10.1002/jcsm.12808

**Published:** 2021-10-28

**Authors:** Gretchen A. Meyer, Karen C. Shen

**Affiliations:** ^1^ Program in Physical Therapy Washington University St. Louis MO USA; ^2^ Departments of Orthopaedic Surgery, Neurology and Biomedical Engineering Washington University St. Louis MO USA

**Keywords:** Dynapenia, Intramuscular adipose tissue, IMAT, Fibro‐adipogenic progenitors, FAP, Female

## Abstract

**Background:**

In response to chronic injury, the muscles of the rotator cuff (RC) experience a unique degeneration characterized by extensive fatty infiltration and loss of contractile function. Human studies suggest this degeneration is also a feature of RC sarcopenia and may precede RC injury. In this study, we investigated whether RC muscles exhibit a similar unique sarcopenia in the mouse.

**Methods:**

Male and female mice were subdivided into four age groups: 3, 9, 18, and 24 months. The supraspinatus (SS) and infraspinatus muscles of the RC and the tibialis anterior (TA) muscle of the hindlimb were assessed. Muscle mass, contractile function, fibre cross‐sectional areas and numbers, fatty infiltration, and fibrosis were assessed at each time point. Targeted transcriptional analyses were performed to assess the role of metabolic and inflammatory derangement in the pathology.

**Results:**

The 24‐month‐aged female mice exhibited decreased mass (25% lower than at 9 and 18 months, *P* < 0.01) in all muscles tested. However, only RC muscles also exhibited decreased contractile tension at this time point (20% lower than at 18 months, *P* < 0.005). Similarly, only female RC muscles exhibited increased fatty infiltration at 24 months (20% higher than 9 months, *P* < 0.05) and had elevated transcriptional markers of adipogenesis (2.4‐fold higher Pparg and 3.8‐fold higher Adipoq expression compared with 9 months, *P* < 0.001). Unbiased metabolic transcriptional profiling identified up‐regulation of the antigen presentation (*Z* scores of 2.3 and 1.9 for SS and TA, respectively) and cytokine and chemokine signalling (*Z* scores of 3.1 and 2.4 for SS and TA, respectively) pathways in 24 month female muscle compared with 9. Further transcriptional analysis supported increased expression of pro‐adipogenic inflammatory signals (6.3‐fold increase in Il6 and 5.0‐fold increase in Anxa2, *P* < 0.01) and increased presence of fibro‐adipogenic progenitors (2.5‐fold) in the 24‐month‐aged female RC compared with 9 months that together exacerbate fatty infiltration.

**Conclusions:**

These data indicate that female mice replicate the unique sarcopenic pathology in the ageing human RC. Furthermore, they suggest that the exacerbated fatty infiltration is due to an interaction between higher resident fibro‐adipogenic progenitor numbers and an elevated systemic inflammation in aged female mice. We conclude that female mouse RC muscle is a novel system to study both human RC degeneration and the signals that regulate sarcopenic fatty infiltration in general, which is prevalent in humans but largely absent from the rodent hindlimb.

## Introduction

Rotator cuff (RC) injury and functional limitations of the shoulder are prevalent in the aged population, with individuals over 60 being five times more likely to develop a symptomatic tear.^1^ Furthermore, the aged shoulder is difficult to rehabilitate, with individuals over 60 facing quadruple the risk for retear following surgical repair^2^ and triple the likelihood of retaining a functional deficit following 6 months of intensive rehabilitation.^3^ These relatively poor outcomes arise in part from a unique degenerative muscle pathology in the aged RC.^4^ Specifically, appearance of ectopic adipose tissue in muscles (fatty infiltration) is considered a hallmark of RC muscle degeneration and is highly correlated with retear and poor functional recovery.^5^, ^6^ Furthermore, fatty infiltration increases in RC muscle with age even in absence of a tear.^7^, ^8^ This suggests that muscle degeneration during ageing could act in a positive feedback loop, both contributing to the high incidence of RC tears and poorer outcomes with age.

Interestingly, there is significant variability in the degree of atrophy and fatty infiltration within an age and injury category,^8^, ^9^ suggesting there are additional factors that determine the progression of muscle degeneration. One of these factors may be intrinsic muscle susceptibility. While fatty infiltration is not unique to RC injury, evidence suggests that it is exacerbated there. In comparable chronic tears to the Achilles tendon, fatty infiltration of the gastrocnemius muscle rarely reaches 50% of the muscle volume,^10^, ^11^ while in chronic RC tears, supraspinatus fatty infiltration frequently exceeds 50%.^9^, ^12^ Studies in rodents support a muscle specificity in injury‐induced pathology—specifically noting increased fatty infiltration in RC muscles compared with muscles of the hindlimb.^13^, ^14^ Even within the RC, there appears to be unique degenerative presentations between muscles, with the supraspinatus and infraspinatus being 2–3 times more likely than the teres minor to exhibit measurable fatty infiltration with age and injury.^8^

Sex may also play a role in susceptibility to muscle degeneration. RC fatty infiltration, independent of atrophy, is worse in women than in men,^6^, ^7^ and this is likely related to the greater functional deficit that women report for common shoulder diagnoses.^15^ Interestingly, this does not appear to be a global feature of female sarcopenia, as fatty infiltration in the lower body with age is reported to be higher in men.^16^ Together, this highlights the need to better understand the interplay between age, anatomy, and sex in the progression of sarcopenia.

In this study, we sought to determine whether the anatomical and sex specificity for fatty infiltration in human sarcopenia was mimicked in the mouse and to investigate which mechanisms contribute to it. We found that muscles of the mouse rotator cuff and hindlimb experience a similar degree of atrophy with age, but only female rotator cuff muscles exhibit a loss of specific contractile force (dynapenia) with exacerbated fatty infiltration. We hypothesize that this specificity is driven by higher numbers of fibro‐adipogenic progenitor cells resident in rotator cuff muscles combined with elevated systemic inflammation in female mice.

## Methods

### Animals and experimental design

Experiments were performed on male and female C57BL/6J mice (Jackson Laboratory, Bar Harbor, ME, USA) subdivided into four age groups—3, 9, 18, and 24 months—with *n* = 5 mice per group. Mice were housed in standard 22°C conditions, allowed free cage activity and *ad libitum* access to food and water. Three muscles were assessed from each mouse, two from the rotator cuff—supraspinatus (SS) and infraspinatus (IS)—and one from the hindlimb—tibialis anterior (TA). Muscles were harvested bilaterally, with one side allocated to contractile testing and to quantification of fatty infiltration and the other side to histological and transcriptional analyses. All procedures were carried out in accordance with the National Institutes of Health's guidelines, as approved by Washington University School of Medicine's Animal Studies Committee.

Methods are presented in summary. Additional methodological detail is provided in *Data* S1.

### Contractile testing

The SS muscle contractile function was assessed as previously described.^17^ For this study, this procedure was adapted to assess the contractile function of the IS and TA as well. Briefly, bony muscle/tendon attachments were left intact and anchored to a custom *ex‐vivo* physiology rig in a bath of Mammalian Ringers. Stimulation was elicited by parallel plate electrodes and force recorded with a dual mode ergometer. Twitch and tetanic contractions were recorded at optimal length and forces were normalized by physiological cross‐sectional area (PCSA).

### Quantification of fatty infiltration

Following contractile testing, IMAT content was quantified as previously described.^18^ Briefly, muscles were decellularized in 1% solution of sodium dodecyl sulfate, fixed in 3.7% formaldehyde and stained with Oil Red O. Fatty infiltration was quantified by reading the optical density of alcohol extracted lipid.

### Histological analyses

Muscles dissected for histological analysis were flash frozen in liquid nitrogen‐cooled isopentane. All muscles were cut at the mid‐belly into 10 μm sections. Fresh sections were immunostained for myosin heavy chain isoforms and laminin or picrosirius red. Details of antibodies and quantification can be found in the Supporting information *Data* S1.

### Transcriptional analyses

A portion of the proximal third of each SS and TA muscle from the 9 and 24 month old groups was saved for gene expression analysis. RNA was purified using a combination Trizol/Chloroform extraction and RNEasy kit with DNAse treatment (Qiagen; Hilden, Germany) as per manufacturer's instructions. Gene expression profiles of 748 genes were analysed using the Nanostring ‘nCounter XT Codeset Gene Expression Assays’ protocol by the Genome Technology Access Center (Washington University, St. Louis, MO, USA). Remaining RNA was used to assay transcription of genes of interest that were not included in the Metabolic Pathways panel by quantitative PCR. A signalling network including infiltrating immune cells, fibro‐adipogenic progenitors (FAPs), atrophic fibres, and activated satellite cells was constructed based on published muscle‐specific pathways.^19^

### Flow cytometry

Quantification of fibro‐adipogenic progenitors was performed via flow cytometry as previously described.^20^ FAPs were identified as the percentage of CD45/CD31 single cells positive for Sca‐1.

### Statistical analyses

Comprehensive grouped data (inclusive of all ages) were analysed by two‐way analysis of variance (ANOVA) for each muscle independently. Significant comparisons between age groups within a sex were determined by Sidak's multiple comparisons test. Subset grouped data (9 and 24 month ages; TA and SS only) were analysed by three‐way ANOVA using muscles taken from the same mouse as a repeated measure. Significant comparisons between groups that differ by only one factor were determined by Sidak's multiple comparisons test. All data sets were tested for normality by the Shapiro–Wilk test and for homogeneity of variance by Levene's test. Significance was set at *P* < 0.05. All analyses were performed using GraphPad Prism 6 (GraphPad Software Incorporated, La Jolla, CA, USA). All data are expressed as mean ± standard deviation.

## Results

### Loss of muscle mass with age varies by sex

**Figure 1:**
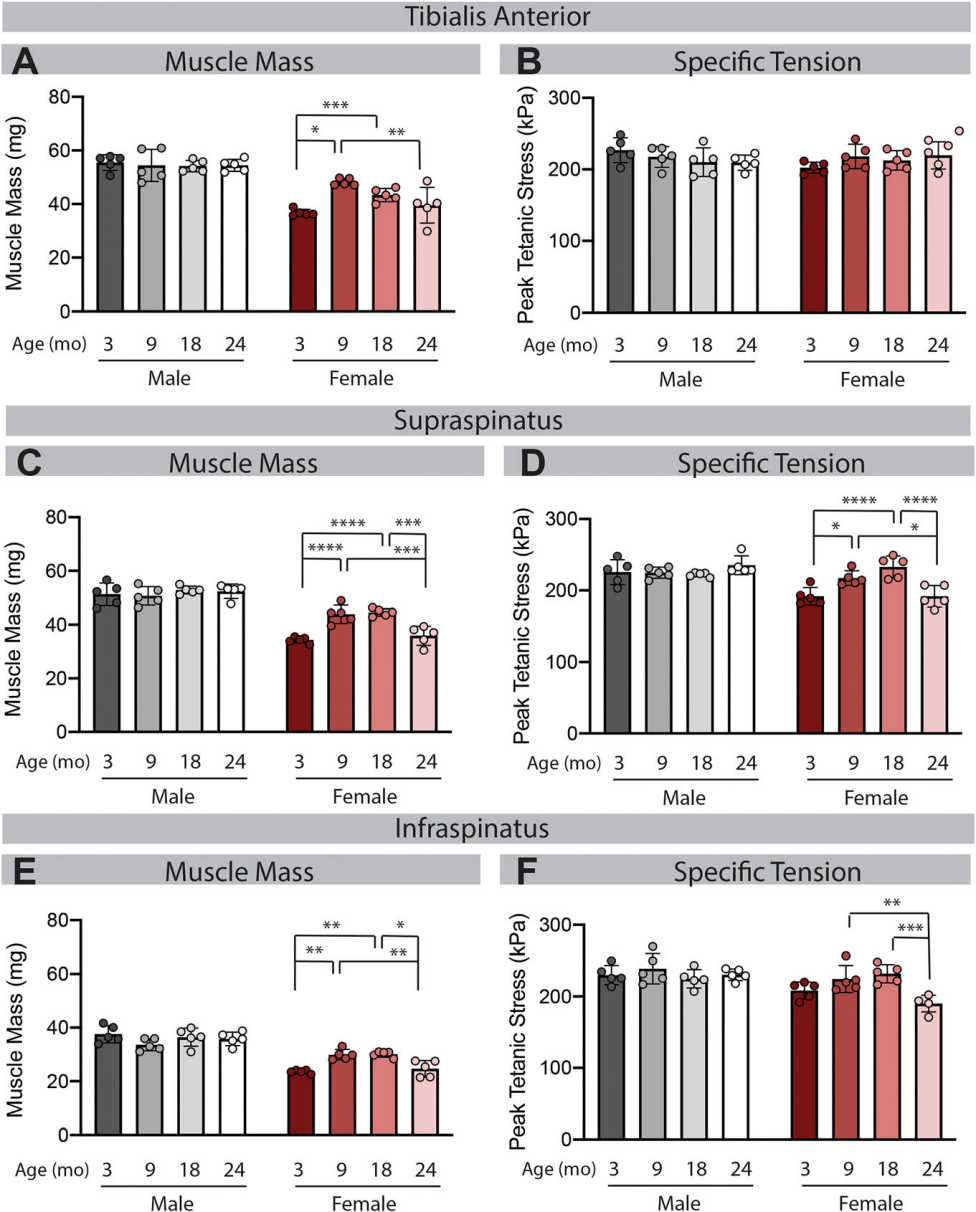
Loss of muscle mass is accompanied by reduced specific tension in the female rotator cuff. In male mice, none of the muscles studied decreased mass or specific tension with age (grey bars). In female mice, all muscles studied significantly decreased mass with age (*A*, *C*, and *E*; red bars). Despite similar changes in mass, only the supraspinatus (SS) and infraspinatus (IS) muscles had a significant decrease in specific tension at 24 months (*B*, *D*, and *F*; red bars). Two‐way ANOVA of Parts (*A*, *C*, *E*, *D*, and *F*) finds a significant effect of sex and age and a significant sex‐age interaction term owing to the variation in mass and specific tension with age in female mice. In Part (*B*), there were no significant effects or interactions. *P < 0.05, **P < 0.01, ***P < 0.005, ****P < 0.001.

All mice in this study were overtly healthy without appearance of dramatic wasting or obesity. Body weight increased with age in both sexes until 18 months where it remained stable in male mice through 24 months and significantly decreased in female mice (*Table* S1). Mass of the TA, SS, and IS muscles all remained stable across age groups in male mice—even at 24 months (*Figure* 1A, 1C, and 1E). In female mice, muscle mass increased from 3 to 9 months, then decreased approximately 25% between 9 and 24 months across all muscles (*Figure* 1A, 1C, and 1E). In the female TA (*Figure* 1A), mass began to decline at 18 months and was not significantly different between 18 and 24 months, while in the SS (*Figure* 1C) and IS (*Figure* 1E) mass remained stable from 9 to 18 months and then was significantly different between 18 and 24 months.

### Loss of muscle contractile function with age varies by sex and muscle

Raw values for peak tetanic tension trended similarly to respective muscle masses, as would be expected due to the strong correlation between mass and tetanic tension in healthy muscle. There was no significant decline in tension with age in any male muscle or the female TA (*Table* S1). However, there were significant declines in tension between 18 and 24 months in both female SS and female IS (*Table* S1). Importantly, accounting for the differences in muscle size by normalizing the raw tension values to the muscle PCSA was unable to explain the deficit. Specific tension (tension per unit PCSA) was significantly lower in female 24 month IS and SS compared with 9 and 18 months (*Figure* 1D and 1F). Thus, the two muscles of the rotator cuff exhibited a unique loss of specific tension (a.k.a. dynapenia), despite a similar loss of mass compared with the TA.

### Loss of muscle mass in female mice is associated with fast fibre atrophy and not hypoplasia

**Figure 2:**
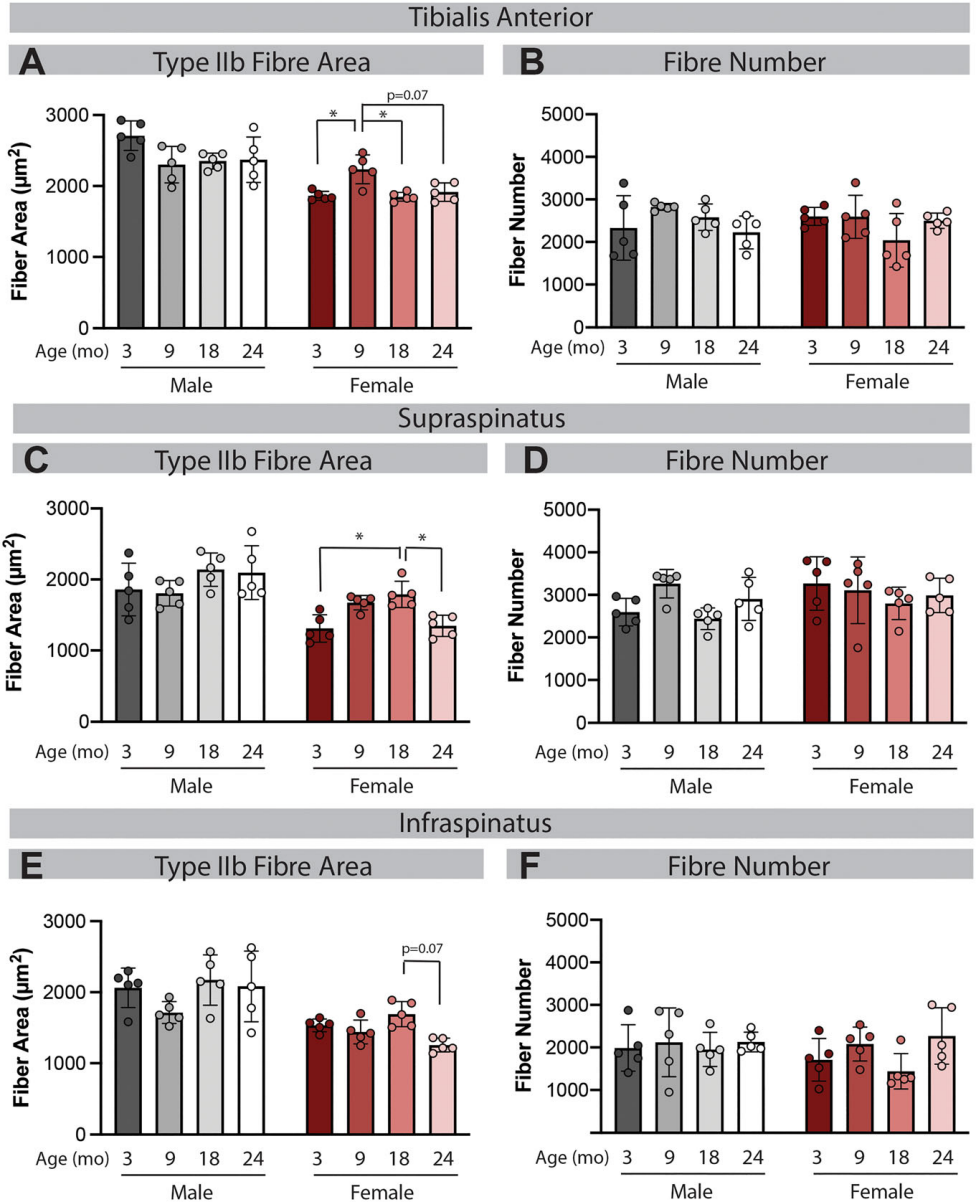
Female muscle exhibits fast fibre atrophy but not hypoplasia. In line with mass changes, there is no difference in cross‐sectional areas of Type IIb fibres or total fibre numbers in any male muscle (grey bars). In the female tibialis anterior (TA), there is a trend for a decrease in Type IIb fibre area at 24 months compared with 9 (*A*; red bars). In the female supraspinatus (SS), there is a significant decrease in Type IIb area at 24 months compared with 18 (*C*; red bars) and a trend towards the same in the infraspinatus (IS) (*E*; red bars). Two‐way ANOVA of Parts (*A*, *C*, and *E*) finds a main effect of sex in all muscles, a main effect of age in the SS and IS and a significant age–sex interaction in the TA and SS. No significant main effects, interactions, or group comparisons were found for total fibre numbers (*B*, *D*, and *F*). *P < 0.05.

To determine whether the loss of muscle mass in female mice was due to a decrease in cross‐sectional area (CSA) of existing fibres, a decrease in fibre number or a combination, fibre CSA, and numbers were assessed on histological sections. In line with muscle mass, there was no decrease in fibre CSA with age in male mice for any fibre type or any muscle (*Figure* 2A, 2C, and 2E; *Table* S2). In female mice, there was an approximately 20% decrease in Type IIb CSA between 9 and 24 months in the TA, and between 18 and 24 months in the SS and IS (*Figure* 2A, 2C, and 2E). Decreases in CSA at 24 months were noted for Types IIa and IIx fibres as well, but these rarely reached significance (*Table* S2). By contrast, there were no significant changes in fibre number with age in any muscle from either sex (*Figure* 2B, 2D, and 2F). As the TA, SS, and IS muscles are all predominantly Type IIb fast fibres, these data suggest that fibre atrophy, not hypoplasia, is the main driver of loss of muscle mass with ageing in female mice. Additionally, the TA was the only muscle to exhibit differences in fibre type distribution with age (*Table* S2) suggesting that fibre type shifts are not driving dynapenia in the female rotator cuff.

### Fatty infiltration increases with age in the female rotator cuff

**Figure 3:**
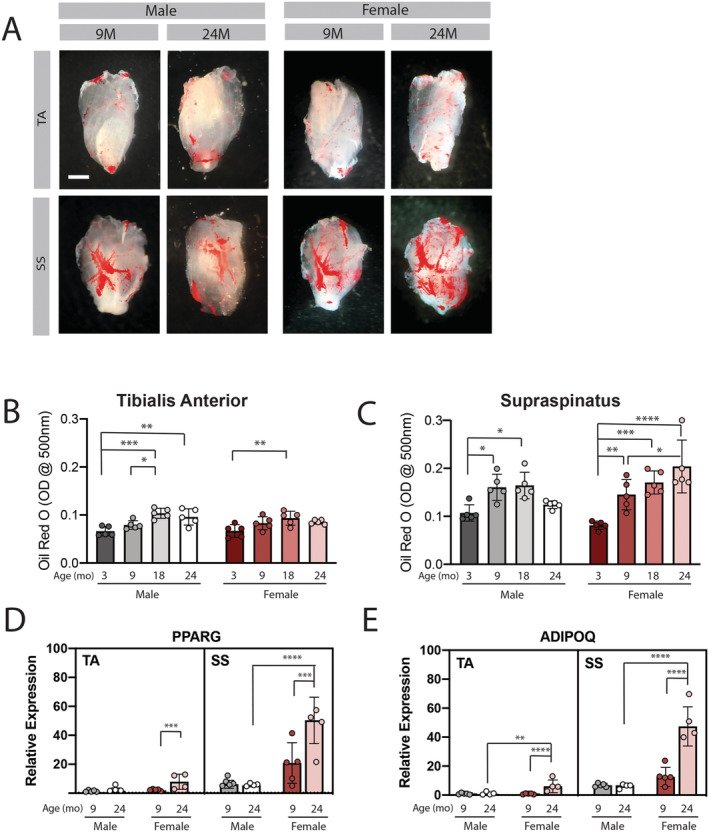
The supraspinatus muscle of female mice experiences progressive fatty infiltration with age. Representative images of Oil Red O (ORO) stained decellularized muscles illustrate both the increased intramuscular adipose tissue in the supraspinatus (SS) compared with the tibialis anterior (TA) at 9 months and its progressive expansion in the female SS at 24 months (*A*; red areas). Quantification of extracted ORO shows an increase in intramuscular adipose during development (from 3 to 18 months) in both muscles and a continued increase in the female supraspinatus at 24 months (*B* and *C*). Two‐way ANOVA of Parts (*B* and *C*) finds a significant main effect of age in both the TA and SS, but only a significant age‐sex interaction in the SS. Transcriptional expression of early (PPARG; *D*) and late (ADIPOQ; *E*) markers of adipogenesis show significant up‐regulation with age in both female TA and SS, with a larger fold change in the SS. Three‐way ANOVA of Parts (*D* and *E*) found a significant effect of muscle, age and sex with significant sex–muscle, sex–age, muscle–age, and sex–muscle–age interactions. *P < 0.05, **P < 0.01, ***P < 0.005, ****P < 0.001.

To further probe the loss of specific force production in the 24 month female rotator cuff muscles, we assessed whether these muscles were experiencing pathological replacement of contractile material. Due to the similarity between the SS and IS muscles, only SS data are compared with TA from this point forward, with available IS data presented in the supporting information. Replacement by intramuscular adipose tissue (a.k.a. fatty infiltration) was assessed in decellularized muscles by Oil Red O staining. Qualitatively, the SS contains more adipose tissue than the TA, which is focused around a central vessel branch (*Figure* 3A; red staining). While male SS exhibits a similar pattern of adipose distribution at 24 months as at 9, the female SS exhibits expansion of adipose beyond the vessel boundary into the muscle bulk (*Figure* 3A). Quantification of this staining by alcohol extraction confirms these qualitative observations. Both muscles increase intramuscular adipose during development to adulthood (from 3 to 9 and/or 18 months), but only in the female SS does this increase continue out to 24 months (*Figure* 3B and 3C). Oil Red O staining of the IS resembled the SS in both qualitative distribution and quantification (*Figure* S1). Assessment of transcriptional markers of adipogenesis supports the morphological findings. Peroxisome proliferator‐activated receptor gamma (Pparg), an early marker of adipogenesis, is more than two‐fold increased at 24 months compared with 9 months in female muscles only (*Figure* 3D). Furthermore, expression of Pparg was nearly 10‐fold higher in the 24 month female SS compared with the 24 month male SS. Similarly, expression of adiponectin (Adipoq), a transcriptional marker of mature adipocytes, is increased more than three‐fold at 24 months compared with 9 months only in female muscles, with a 7‐fold increase in 24 month female SS and TA compared with respective male muscle (*Figure* 3E). Taken together, these data illustrate an exacerbated fatty infiltration in female muscles with age that is more dramatic in the muscles of the rotator cuff.

### Fibrosis increases with age in the female rotator cuff

**Figure 4:**
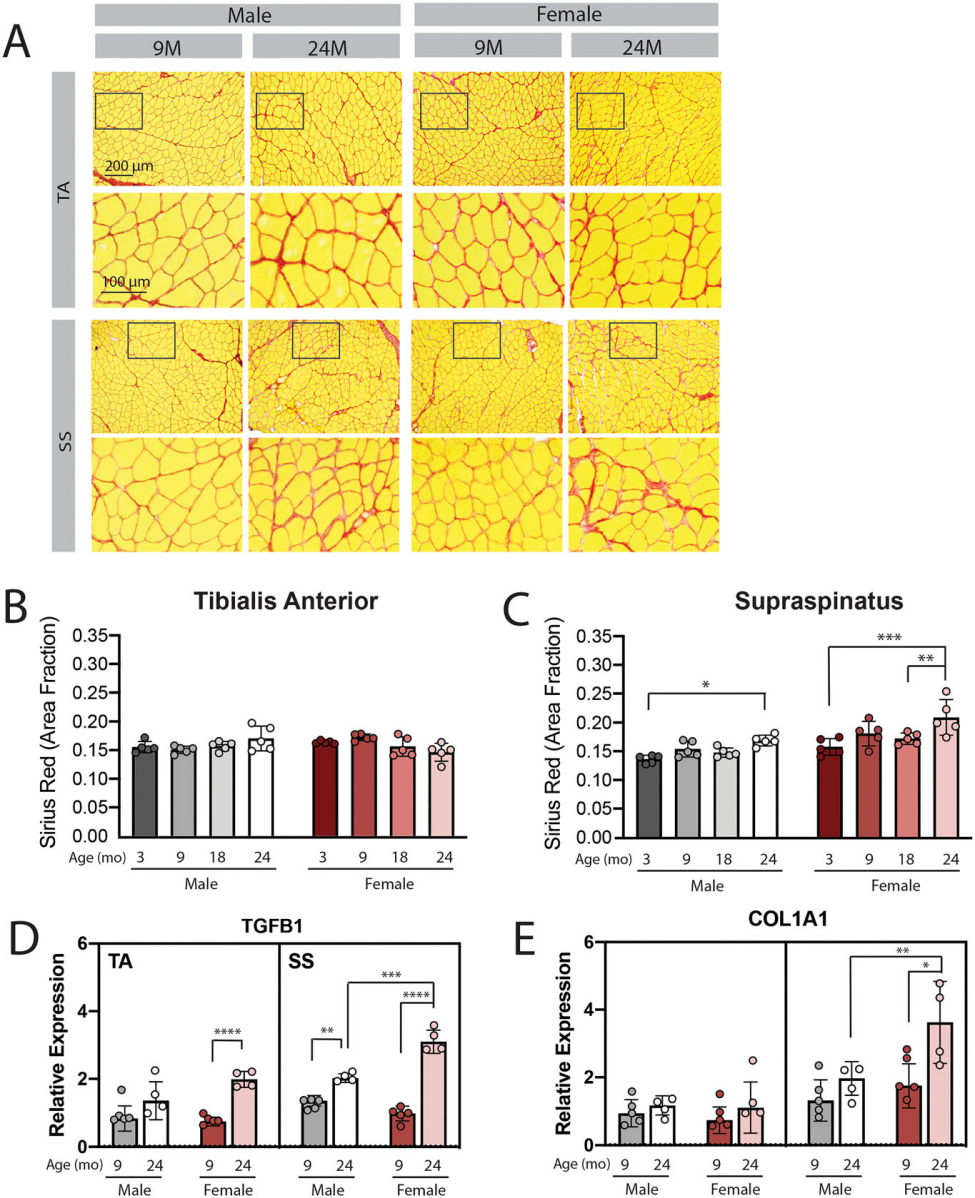
The supraspinatus muscle of both sexes experiences fibrosis with age. Representative images of Sirius Red stained histological sections (A upper rows; red areas) illustrate focal areas of increased extracellular matrix (A lower rows; magnified insets) in the 24 month supraspinatus (SS) compared with 9 month in both male and female mice. Quantification of the Sirius Red stained area fraction shows a significant increase in matrix at 24 months in both male and female SS muscles, with no difference in the tibialis anterior TAs (*B* and *C*). Two‐way ANOVA found significant main effects of gender and age in the SS, but no significant interaction. The fibrotic regulator gene TGFB1 was significantly up‐regulated with age in female TA and SS and male SS (*D*). Expression of the matrix component COL1A1 was significantly increased in female SS only (*E*). Analysis of Parts (*D* and *E*) by three‐way ANOVA found a significant effect of muscle, age, and sex with significant sex–muscle, sex–age, and muscle–age interactions for both TGFB1 and COL1A1. *P < 0.05, **P < 0.01, ***P < 0.005, ****P < 0.001.

To determine whether expansion of connective tissue (a.k.a. fibrosis) is also exacerbated in aged muscles of the female rotator cuff, histological sections were stained with Sirius Red. Qualitatively, areas of local collagen expansion could be found in all muscles at 24 months compared with 9 months (*Figure* 4A; red in magnified regions). When quantified over the majority of the muscle section, there was no significant change in the area fraction of Sirius Red staining in the TA with age in either sex. However, there was a small, but significant, increase in area fraction from 3 to 24 months in the SS in both sexes and a significant increase in the female SS from 18 to 24 months (*Figure* 4C). Expression of transforming growth factor beta (Tgfb1), a master regulator of the fibrogenic response, was elevated with age in all muscles except the male TA (*Figure* 4D). The increase was greatest in the female SS, where expression was three‐fold higher at 24 months than at 9 months. Expression of the alpha 1 chain of collagen 1 (Col1a1), a major component of skeletal muscle extracellular matrix, was significantly increased with age in the female SS only (*Figure* 4E). These data, combined with *Figure* 3, support an exacerbated fibro‐fatty replacement of contractile material in the aged female rotator cuff muscles that could contribute to dynapenia by decreasing muscle quality.

### Aged female muscles have altered expression of genes related to inflammation and cytokine signalling

**Figure 5:**
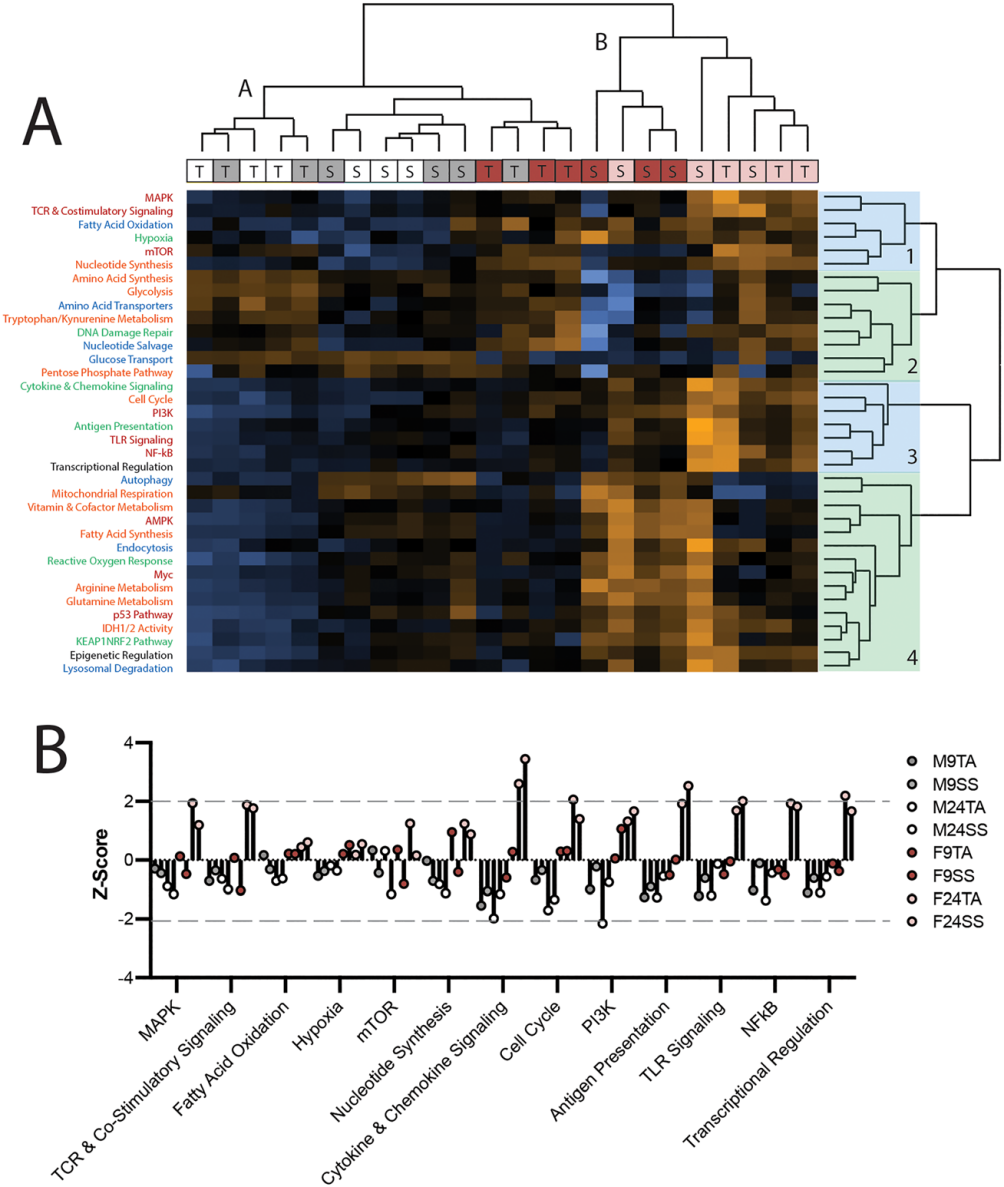
Metabolic transcriptional profiling reveals increased presence of antigen presenting immune cells and inflammatory cytokines in aged female muscle. Unbiased hierarchical clustering by sample and annotated pathway based on the expression of 768 genes included in the NanoString Metabolic Pathways Panel (*A*; heatmap of Z‐scores). The majority of female muscles cluster by age while 24 and 9 month male muscles are intermixed (*A*, top). Pathways divide into four main clusters (*A*, right) that either reflect transcriptional differences between SS and TA (2 and 4, green) or unique regulation in aged female muscles (1 and 3, blue). Pathways are colour coded by category (red: metabolic signalling; blue: nutrient capture and catabolic pathways; orange: biosynthesis and anabolic pathways; green: cell stress; black: transcriptional regulation). *Z*‐scores for pathways that clustered by unique regulation in aged female muscles (*B*).

Because of the mounting evidence that ageing affects mitochondrial function and metabolic signalling, we sought to probe these pathways in a subset of remaining samples through the NanoString metabolic pathways panel. Hierarchical clustering across all 36 pathways grouped samples into two primary clusters (*Figure* 5A). Cluster A contained all the male muscles and female 9 month TA muscles, and Cluster B contained the female 9 month SS muscles and all female 24 month muscles. In cluster A, the male muscles entirely subclustered by muscle (TA vs. SS), while in Cluster B, the female muscles primarily subclustered by age (9 vs. 24 months), suggesting that age was a larger determinant of metabolic pathways in female muscle than in male. Hierarchical clustering by pathway grouped pathways into four clusters. Clusters 1 and 3 were qualitatively distinguished by high *Z*‐scores in the female 24 month muscles. Cluster 2 was qualitatively distinguished by high *Z*‐scores in TA muscles, and Cluster 4 was distinguished by high *Z*‐scores in SS muscles.

Surprisingly, despite their different specific force production capacity and fibro/fatty replacement, the 24 month female SS muscles did not cluster separately from the 24 month female TA, and none of the metabolic pathways appeared to be specifically differentially regulated in this group. Also somewhat surprisingly, the traditional metabolic pathways (orange in *Figure* 5A) nearly exclusively clustered in 2 and 4, suggesting that there are marked differences in metabolism between the TA and SS, but these differences are largely unaffected by ageing.

To further probe which pathways were differentially regulated in the 24 month female samples in general, we grouped samples and assessed *Z*‐scores in the pathways of Clusters 1 and 3. As expected, 24 month female TA and SS muscles trended together in these pathways and had a *Z*‐score at or above 2 in 8 of the 13 pathways (*Figure* 5B). Five of these eight can be tied to inflammation in muscle: TCR and Co‐stimulatory signalling, cytokine and chemokine signalling, antigen presentation, TLR signalling, and NFkB.

### Increased inflammatory regulation of fibro/adipogenic progenitor cells may drive fibro‐fatty replacement in the aged female rotator cuff

**Figure 6:**
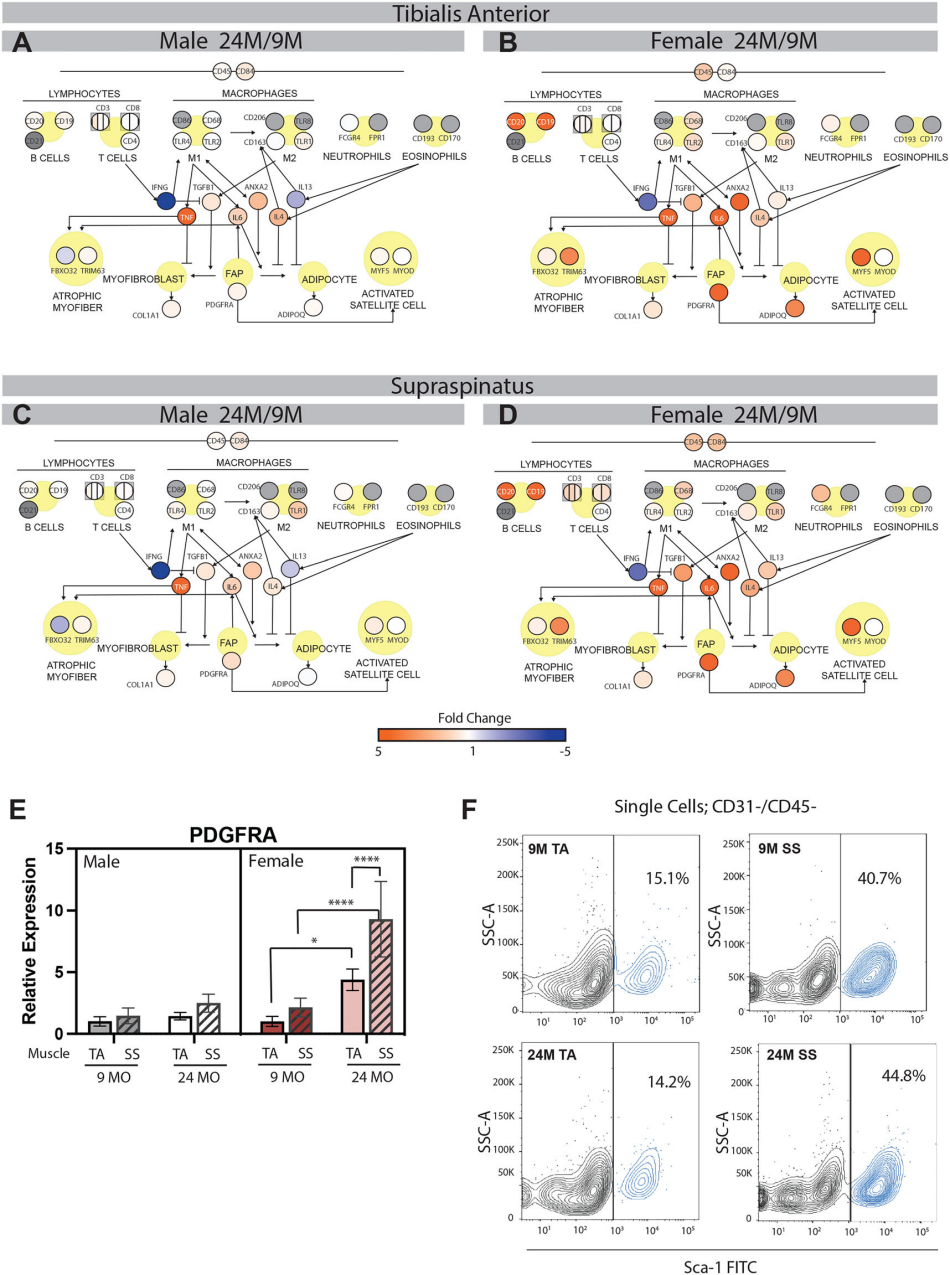
Aged female muscle has increased expression of inflammatory cytokines involved in fibro‐adipogenic progenitor (FAP) signalling. A diagram of the action of select inflammatory cytokines on FAPs, myofibres, and satellite cells pseudocoloured for transcriptional regulation in 24 month muscles compared with 9 (*A–D*). Grey gene circles represent genes that were not assessed as part of the NanoString panel or by qPCR. Predicted action of cytokines on cells and processes is indicated as positive (arrow) or negative (block). Female muscles have elevated expression of immune cell markers and the pro‐adipogenic cytokines IL6 and ANXA2 with age. Expression of PDGFRA, a marker of undifferentiated FAPs, is significantly increased with age in both the female TA and SS, with a significantly greater expression in the aged SS compared with TA (*E*). Flow cytometry identifies an increased fraction of CD31‐/CD45‐/Sca‐1+ cells in the female SS at both 9 and 24 months (*F*). *P < 0.05, ****P < 0.001.

Inflammatory cytokines play a regulatory role in the fibrogenic or adipogenic fate determination of FAPs, driving them to contribute to fibrosis and fatty infiltration in muscle injury and disease.^21^ To illustrate the potential roles of these cytokines, we have overlayed gene expression data on a pathway map involving cell surface markers of immune cells (part of the NanoString panel), regulatory cytokines (qPCR) and transcriptional markers of FAPs, myofibroblasts, adipocytes, atrophic myofibres and activated satellite cells (qPCR) (*Figure* 6A–6D). Overall, there was a greater increase in expression of immune cell markers in female muscle with age than in male. These include pan‐haematopoietic (CD45 and CD68), B‐cell and T‐cell, both classically activated (M1) and alternatively activated (M2) macrophages and neutrophils. In line with this, there was also higher expression of many FAP regulatory cytokines these cells are thought to secrete. Specifically, relative expression of tumour necrosis factor alpha (Tnf), Tgfb1, interleukin 6 (Il6), annexin A2 (Anxa2) and interleukin 13 (Il13) was more than doubled in female mice compared with male mice (*Table* S3). Because some of these cytokines are reported to promote FAP fibrogenesis or adipogenesis (arrows) and some are reported to inhibit them (blocks), it is difficult to pinpoint a directional pressure on FAPs from this inflammatory environment. And indeed, there is transcriptional evidence for both elevated fibrogenesis and adipogenesis with age in female muscles, marked by Col1a1 and Adipoq in this pathway. Undifferentiated FAPs (marked by platelet derived growth factor alpha (Pdgfra) expression) may play a role in guiding the behaviour of activated satellite cells. Increased expression of myogenic factor 5 (Myf5) in aged female muscles suggests this interaction could be occurring as well. Additionally, inflammatory cytokines also have direct action of muscle fibres and can activate atrophic signalling. Expression of two major ubiquitin ligases involved in muscle atrophy, atrogin 1 (Mafbx; Fbxo32), and muscle ring finger protein 1 (Murf1; Trim63), which are targets of TNFα and IL6, are increased with age in female muscle more dramatically than in male muscle.

Interestingly, while this evidence supports an exacerbated inflammatory environment in aged female muscle, it appears similar between the SS and the TA and thus is not a good explanator of the differential fatty infiltration and/or specific force deficit between these muscles. We hypothesized that elevated systemic inflammation in the aged female mice may be interacting with intrinsic differences between the SS and TA to contribute to these effects—specifically differences in FAP numbers at baseline. Expression of Pdgfra was significantly approximately 2.5‐fold elevated in 24 month female SS compared with TA, suggesting either higher Pdgfra expression by FAPs or a greater number of FAPs in the SS (*Figure* 6E). To differentiate between the two, we assessed the percentage of PDGFRα positive cells in muscles from our last 24 month female paired with a 9 month female. While the data are only from a single muscle per group, we do find an approximately 2.5‐fold elevated percentage of Sca‐1 positive cells in both the 9 and 24 month SS compared with the TA (*Figure* 6F). This is consistent with previously published findings of increased FAPs in the muscles of the rotator cuff^14^ and suggests that the rotator cuff may be primed for fibro‐fatty replacement in an inflammatory environment.

## Discussion

In this study, we show a unique sarcopenic pathology in the female mouse rotator cuff characterized by a loss of specific force production (dynapenia) accompanied by fatty infiltration. These features were both sex‐specific and muscle‐specific as they were not significantly altered in either the male RC or the female hindlimb. The sex‐specificity of fatty infiltration mimics human RC sarcopenia^7^ and suggests that aged female mice could shed light on the differential outcomes in the treatment of chronic rotator cuff disease in women.^15^ The muscle‐specificity grossly mimics general anatomical differences in human sarcopenia, although it is more difficult to divide this comparison by sex. No single study compares features of sarcopenia in the RC to the lower limb, but comparison between studies shows an approximately five‐fold increase in thigh IMAT in the upper quartile of 60–80 year olds relative to the average of 20–30 year olds^22^ compared with a >10‐fold increase with the same comparison in the RC,^7^ suggesting that age‐related fatty infiltration is exacerbated in the human rotator cuff.

However, other features of human sarcopenia were not apparent in our study. Most striking was the absence of mass loss in male muscles with age. The majority of studies investigating features of sarcopenia in the mouse hindlimb find a 10–20% mass decrease in the TA, gastrocnemius or extensor digitorum longus mass (selected^23^, ^24^), while a only a few find no change.^25^, ^26^ However, a number of studies were unable to attribute the loss of mass to either a reduction in fibre CSA or a decrease in fibre number.^23^, ^27^, ^28^ We did find a reduction in the mass of the TA in female mice, which is consistent with some studies in the female hindlimb but inconsistent with others.^27^, ^29^ Of the studies that compared male to female hindlimb sarcopenia, one found muscle loss in male mice only and the other found it in female mice only.^27^, ^29^ This variability could be due to different normalizations, different ages at comparison or simply reflect natural variability. Evidence suggests only a fraction of mice at a given age exhibit frailty^30^ and the onset of frailty is likely determined in part by housing conditions subtle within‐strain genetic variation. Furthermore, changes in muscle mass may not reflect the onset of frailty and loss of contractile function (dynapenia) has been proposed as a better indicator of overall functional decline.^31^

In female mice, muscles with loss of mass, we find that only RC muscles exhibit a reduction in specific force production. In this study, we adapted *ex‐vivo* physiology to RC muscles and the TA and, to our knowledge, are the first to report a muscle‐intrinsic contractile deficit in the aged RC. Because the muscles were isolated from the nervous and vascular systems during testing, it is unlikely that these factors directly caused contractile deficits through poor neurotransmission or metabolite insufficiency. However, we cannot rule out the subtler contribution of long‐term partial denervation or ischaemia to fibre structure and function. Mitochondrial dysfunction could also drive contractile deficits in this testing paradigm because it relies on cellular stores of calcium and ATP. We did not directly assess mitochondrial structure, enzyme activity or functional respiration in this study, but it is notable that genes involved in mitochondrial respiration were not consistently altered in the aged female RC samples.

Recently, circulating immune cells and pro‐inflammatory cytokines have been proposed to contribute to ageing by altering the tissue microenvironment and disrupting cellular dynamics—a process coined inflamm‐ageing. In muscle, most of the focus has centred on the satellite cell (SC) with evidence suggesting that the aged microenvironment decreases SC numbers and regenerative potential. However, inducible depletion of SCs does not affect the progression of sarcopenia, suggesting that SC dysfunction does not drive muscle loss or dynapenia in the mouse.^32^ Likewise, we find no difference in MyoD expression and an increase in Myf5 expression in aged female muscle, suggesting that the preferential loss of mass in aged females is not due to a deficit in activation or early differentiation of SCs. FAPs are also sensitive to inflammatory cytokines in their microenvironment (reviewed in Biferali *et al*.^21^). We do find evidence for dysregulation of FAPs in aged female muscle including increased adipogenic and fibrogenic gene expression in the SS and TA, and increased fibrosis and fatty infiltration in the SS. While both fibrosis and fatty infiltration are frequently associated with contractile deficits, we recently showed that fatty infiltration can directly impair contraction.^33^ Because significant fatty infiltration was only found in muscles with dynapenia in this study, it is appealing to hypothesize that they are causally related. However, more work needs to be done in this complex system to explore all plausible mechanisms and the interactions between them. Specifically of note, even at young ages, the SS has more fatty infiltration than the TA, yet has no contractile deficit. Similarly, the young female SS has increased expression of adipogenic genes at 9 months compared with male such that the fold changes with age are similar in male and female mice when normalized to their sex‐matched young control—however, only the female SS exhibits a contractile deficit.

Another important question is what drives the muscle specificity of fatty infiltration in aged female mice. While this is not the first study to suggest that fatty infiltration is exacerbated in the RC compared with the hindlimb^13^, ^14^ or in the aged RC compared with the young,^34^ it is the first to demonstrate differences in absence of an injury stimulus. The few studies that have looked at the natural history of fatty infiltration in the male rodent RC have found no increase with age,^34^, ^35^ similar to what we find in male mice. The fact that injury elicits measurable differences suggests altered sensitivity of aged FAPs to microenvironmental cues or differences in the cues themselves—there is evidence for both hypotheses in mice.^36^, ^37^ Based on our transcriptional analysis, we hypothesize that the aged female microenvironment is similarly altering the FAP microenvironment through inflammatory cytokines. In aged female, but not male, mice we find significantly increased expression of inflammatory cytokines thought to drive adipogenesis in FAPs (Il6 and Anxa2) and increased markers of the immune cells from which they are derived. While these changes are similar in the TA and SS, we find that the SS has more resident FAPs to respond to aberrant stimuli—a result previously reported.^14^ There is also evidence suggesting increased adipogenic potential in FAPs from the RC.^14^ Together, this suggests a larger pool of FAPs in the RC that are primed for adipogenesis in response to inflammatory stimuli. In this study, we find that these stimuli increase more dramatically with age in female mice than in male mice.

In summary, we demonstrate a unique sarcopenic progression in the female mouse rotator cuff that could be used as a model to explore the mechanisms behind the muscle and sex specificity of age‐related fatty infiltration in humans. We also propose a plausible mechanism for this specificity. If increased fatty infiltration of the aged female RC is indeed driven by higher concentrations of FAPs in an exacerbated inflammatory environment, then the aged female RC could be a better model to study human fatty infiltration in general. First is because the expression patterns of cytokines in aged female muscle better mimics the inflammatory profile of human ageing. Serum TNF‐α and IL‐6 are increased in the elderly and associated with sarcopenia and elevated risk for mortality.^38^ Additionally, IFN‐γ expression is lower, and IL‐4 expression is higher in stimulated immune cells of elderly compared with young individuals.^39^ Second, the relative percentage of FAPs in the mouse RC (~40%) is closer to that reported for human muscle (40–75%)^40^ and while the degree of fatty infiltration in the aged female RC is still much less than in humans, the study of the age‐induced driving mechanisms could still shed significant light on the progression of fatty infiltration in human sarcopenia. More work is needed to determine how broadly representative the mouse female rotator cuff is, but identifying mechanisms driving specificity in the mouse are likely to shed light on muscle and sex specificity in human ageing and disease.

## Conflict of interest

The authors have declared that no conflict of interest exists.

## Supporting information


**Table S1.** **Additional Physiology Data**. Changes in body weight, raw tetanic and twitch tensions, time intervals from stimulus to peak twitch tension (Time to Peak Tension) and from peak twitch tension to half full relaxation (Half‐Relax Time). Data are presented as mean ± standard deviation. Data in rows were analyzed by 2‐way ANOVA with significance set at *p* < 0.05. (#) main effect of age, (†) main effect of sex, (&) age‐sex interaction, (a) different from 3 MO, (b) different from 9 MO, (c) different from 18 MO.
**Table S2. Additional Fiber Data**. Changes in fiber areas by fiber types and fiber type distributions. Data are presented as mean ± standard deviation. Data in rows were analyzed by 2‐way ANOVA with significance set at *p* < 0.05. (#) main effect of age, (†) main effect of sex, (&) age‐sex interaction, (a) different from 3 MO, (b) different from 9 MO, (c) different from 18 MO.
**Table S3. Additional Gene Expression Data**. Changes in expression of the indicated genes normalized to the mean of MALE 9 MO TA. Data are presented as mean ± standard deviation. Data in rows were analyzed by 3‐way ANOVA with significance set at *p* < 0.05. (#) main effect of age, (†) main effect of sex, (&) age‐sex interaction, (a) different from matched 9 MO.
**Figure S1. The infraspinatus muscle of female mice exhibits fatty infiltration and fibrosis with age.** Representative images of Oil Red O (ORO) stained decellularized muscles (A; top row) and Sirius Red stained histological sections (A; bottom rows). Quantification of extracted ORO shows an increase in intramuscular adipose in the female infraspinatus at 24 months (B). Quantification of the Sirius Red stained area fraction shows an increase in matrix at 24 months (C). 2‐way ANOVA of B and C finds a significant main effect of age, but only a significant age‐sex interaction for Sirius Red area fraction. * *p* < 0.05, ***p < 0.005, ****P < 0.001.

**Data S1.** Supporting information
