# Supplementary material for: A unique sarcopenic progression in the mouse rotator cuff
Source: J Cachexia Sarcopenia Muscle. 2021 Oct 28;13(1):561–73. doi: 10.1002/jcsm.12808 (PMC8818692; doi:10.1002/jcsm.12808)
Supplement: Supplementary file 1 — Table S1. Additional Physiology Data. Changes in body weight, raw tetanic and twitch tensions, time intervals from stimulus to peak twitch tension (Time to Peak Tension) and from peak twitch tension to half full relaxation (Half‐Relax Time). Data are presented as mean ± standard deviation. Data in rows were analyzed by 2‐way ANOVA with significance set at p < 0.05. (#) main effect of age, (†) main effect of sex, (&) age‐sex interaction, (a) different from 3 MO, (b) different from 9 MO, (c) different from 18 MO. Table S2. Additional Fiber Data. Changes in fiber areas by fiber types and fiber type distributions. Data are presented as mean ± standard deviation. Data in rows were analyzed by 2‐way ANOVA with significance set at p < 0.05. (#) main effect of age, (†) main effect of sex, (&) age‐sex interaction, (a) different from 3 MO, (b) different from 9 MO, (c) different from 18 MO. Table S3. Additional Gene Expression Data. Changes in expression of the indicated genes normalized to the mean of MALE 9 MO TA. Data are presented as mean ± standard deviation. Data in rows were analyzed by 3‐way ANOVA with significance set at p < 0.05. (#) main effect of age, (†) main effect of sex, (&) age‐sex interaction, (a) different from matched 9 MO. Figure S1. The infraspinatus muscle of female mice exhibits fatty infiltration and fibrosis with age. Representative images of Oil Red O (ORO) stained decellularized muscles (A; top row) and Sirius Red stained histological sections (A; bottom rows). Quantification of extracted ORO shows an increase in intramuscular adipose in the female infraspinatus at 24 months (B). Quantification of the Sirius Red stained area fraction shows an increase in matrix at 24 months (C). 2‐way ANOVA of B and C finds a significant main effect of age, but only a significant age‐sex interaction for Sirius Red area fraction. * p < 0.05, ***p < 0.005, ****P < 0.001. [file JCSM-13-561-s002.docx]

**A Unique Sarcopenic Progression in the Mouse Rotator Cuff**

Gretchen A. Meyer and Karen Shen

*Washington University School of Medicine, St. Louis, MO*

meyerg@wustl.edu

|  | MALE | | | |  | FEMALE | | | |  | |
| --- | --- | --- | --- | --- | --- | --- | --- | --- | --- | --- | --- |
|  | 3 MO | 9 MO | 18 MO | 24 MO |  | 3 MO | 9 MO | 18 MO | 24 MO | |  |
| Body Weight (g) | 26.06±0.69 | 29.81±1.9 | 32.25±2.56^a^ | 31.63±2.16^a^ |  | 19.18±0.31 | 24.51±1.04^a^ | 34.19±3.26^ab^ | 25.21±3.76^ac^ | | #†& |
| TA Twitch Tension (N) | 262.6±71.5 | 247.7±47.3 | 278.6±54.3 | 293.4±52.0 |  | 171.6±39.3 | 216.8±57.3 | 218.6±37.7 | 206.7±72.3 | | † |
| TA Tetanic Tension (N) | 133.7±159.4 | 1144.2±161.0 | 1080.9±117.5^a^ | 1094.1±138.4^a^ |  | 912.4±42.4 | 1018.9±50.6 | 875.6±82.6 | 1038.7±203.2 | | †& |
| TA Time to Peak Tension (ms) | 0.011±0.002 | 0.012±0.001 | 0.012±0.002 | 0.011±0.0005 |  | 0.011±0.0012 | 0.013±0.001 | 0.011±0.001 | 0.012±0.0006 | |  |
| TA Half-Relax Time (ms) | 0.008±0.002 | 0.008±0.001 | 0.01±0.003 | 0.01±0.0008 |  | 0.007±0.001 | 0.009±0.003 | 0.01±0.002 | 0.008±0.0011 | |  |
| SS Twitch Tension (N) | 290.3±24.8 | 283.9±45.0 | 313.9±65.6 | 261.6±46.7 |  | 180.6±21.2 | 242.3±37.0^a^ | 268.4±34.6^a^ | 202.1±33.4^c^ | | #†& |
| SS Tetanic Tension (N) | 1688.8±124.0 | 14945.0±111.8 | 1759.5±88.0^b^ | 1641.4±112.5 |  | 1103.7±90.5 | 1400.8±162.3^a^ | 1550.5±144.3^a^ | 1034.9±109.8^bc^ | | #†& |
| SS Time to Peak Tension (ms) | 0.009±0.0004 | 0.011±0.0008^a^ | 0.011±0.001^a^ | 0.01±0.001 |  | 0.008±0.0011 | 0.01±0.0004 | 0.01±0.001 | 0.011±0.001^a^ | | #† |
| SS Half-Relax Time (ms) | 0.006±0.0009 | 0.007±0.002 | 0.007±0.001 | 0.006±0.001 |  | 0.007±0.0007 | 0.006±0.001 | 0.008±0.002 | 0.007±0.000 | |  |
| IS Twitch Tension (N) | 228.3±27.2 | 222.7±51.4 | 241.3±29.5 | 251.1±38.9 |  | 132.3±10.8 | 195.3±21.7 | 224.9±65.7^a^ | 207.1±22.7^a^ | | #† |
| IS Tetanic Tension (N) | 1324.9±137.6 | 1136.2±76.9^a^ | 1143.5±56.7^a^ | 1312.7±132.2^bc^ |  | 849.5±71.9 | 1092.9±74.6^a^ | 1081.2±50.7^a^ | 920.7±71.9^bc^ | | †& |
| IS Time to Peak Tension (ms) | 0.009±0.0007 | 0.01±0.0007 | 0.01±0.0008 | 0.009±0.0004 |  | 0.01±0.001 | 0.01±0.0009 | 0.01±0.0011 | 0.011±0.0004 | | † |
| IS Half-Relax Time (ms) | 0.007±0.001 | 0.007±0.0008 | 0.008±0.0006 | 0.007±0.0008 |  | 0.009±0.002 | 0.008±0.0006 | 0.007±0.001 | 0.01±0.0015^a^ | | † |

**Supplemental Table 1: Additional Physiology Data**. Changes in body weight, raw tetanic and twitch tensions, time intervals from stimulus to peak twitch tension (Time to Peak Tension) and from peak twitch tension to half full relaxation (Half-Relax Time). Data are presented as mean ± standard deviation. Data in rows were analyzed by 2-way ANOVA with significance set at p<0.05. (#) main effect of age, (†) main effect of sex, (&) age-sex interaction, (a) different from 3 MO, (b) different from 9 MO, (c) different from 18 MO.

|  |  | MALE | | | |  | | FEMALE | | | | |  | |
| --- | --- | --- | --- | --- | --- | --- | --- | --- | --- | --- | --- | --- | --- | --- |
|  |  | 3 MO | 9 MO | 18 MO | 24 MO |  | 3 MO | | 9 MO | 18 MO | 24 MO |  | |  |
|  | TA Type 2a Fiber Area (um^2^) | 884.8±189.8 | 1065.2±143.4 | 815.6±125.0 | 735.3±67.6^b^ |  | 812.2±179.1 | | 704.0±130.2 | 764.5±84.7 | 806.3±184.0 | †& | |  |
|  | TA Type 2x Fiber Area (um^2^) | 1439.4±272.7 | 1680.1±207.6 | 1338.2±270.6 | 1190.8±165.5^b^ |  | 1279.5±226.5 | | 1213.9±141.0 | 1247.2±175.2 | 1250.7±159.6 | † | |  |
|  | SS Type 2a Fiber Area (um^2^) | 840.7±125.4 | 899.3±165.6 | 971.4±178.7 | 943.6±109.7 |  | 728.6±57.2 | | 781.9±132.2 | 981.4±131.6^a^ | 953.4±150.4 | # | |  |
|  | SS Type 2x Fiber Area (um^2^) | 1279.1±269.4 | 1296.4±120.1 | 1545.6±327.2 | 1448.1±227.1 |  | 972.9±186.1 | | 1164.2±155.2 | 1368.8±171.3^a^ | 1090.1±219.1 | #† | |  |
|  | IS Type 2a Fiber Area (um^2^) | 857.0±104.5 | 761.4±158.7 | 877.3±98.1 | 831.9±120.9 |  | 837.0±179.5 | | 730.2±161.1 | 751.4±115.7 | 709.4±254.0 |  | |  |
|  | IS Type 2x Fiber Area (um^2^) | 1134.0±203.1 | 1061.3±92.3 | 1321.4±236.2 | 1229.2±126.2 |  | 1096.5±140.9 | | 968.4±90.6 | 1456.3±478.0^b^ | 1062.3±302.7 | # | |  |
| Superficial | TA Type 2a Fiber Percentage (%) | 0.24±0.53 | 0.00±0.00 | 0.74±1.20 | 1.18±2.63 |  | 2.33±3.23 | | 0.85±0.93 | 5.41±5.44^b^ | 0.24±0.54^c^ | † | |  |
|  | TA Type 2x Fiber Percentage (%) | 21.71±7.41 | 25.12±6.07 | 18.74±10.03 | 17.57±10.23 |  | 19.90±7.95 | | 22.05±7.57 | 27.17±7.17 | 10.33±5.04^c^ | # | |  |
|  | TA Type 2b Fiber Percentage (%) | 78.05±7.17 | 74.71±5.85 | 80.51±10.36 | 81.26±12.30 |  | 77.36±8.21 | | 76.72±7.27 | 67.25±9.33 | 89.43±5.17^c^ | # | |  |
|  | SS Type 2a Fiber Percentage (%) | 5.49±5.76 | 1.54±1.31 | 2.47±2.14 | 6.17±2.54 |  | 5.86±3.74 | | 9.62±7.41 | 5.42±4.62 | 8.66±6.61 | † | |  |
|  | SS Type 2x Fiber Percentage (%) | 18.16±4.97 | 27.43±8.74 | 21.30±4.10 | 22.69±8.44 |  | 16.32±4.76 | | 20.05±2.08 | 27.05±6.99 | 16.77±7.00 |  | |  |
|  | SS Type 2b Fiber Percentage (%) | 76.35±4.34 | 71.02±9.93 | 76.09±3.55 | 71.64±10.31 |  | 77.90±3.89 | | 70.87±7.13 | 67.53±7.94 | 74.97±5.79 |  | |  |
|  | IS Type 2a Fiber Percentage (%) | 6.18±1.93 | 8.90±1.15 | 7.25±4.22 | 10.56±6.09 |  | 8.04±1.86 | | 7.27±2.94 | 10.08±6.06 | 12.15±1.99 |  | |  |
|  | IS Type 2x Fiber Percentage (%) | 18.76±4.92 | 19.74±5.61 | 20.42±10.1 | 28.37±11.19 |  | 18.39±7.95 | | 20.94±7.16 | 20.52±8.88 | 23.59±7.73 |  | |  |
|  | IS Type 2b Fiber Percentage (%) | 75.05±5.47 | 71.36±5.97 | 72.33±12.21 | 61.07±12.37 |  | 74.09±7.24 | | 71.79±4.62 | 69.18±12.78 | 65.31±10.07 |  | |  |
| Deep | TA Type 1 Fiber Percentage (%) | 0.00±0.00 | 0.00±0.00 | 0.00±0.00 | 0.00±0.00 |  | 0.23±0.51 | | 1.00±1.41 | 0.20±0.45 | 1.00±0.71 | † | |  |
|  | TA Type 2a Fiber Percentage (%) | 20.02±8.24 | 26.19±8.28 | 20.66±4.72 | 14.09±5.90 |  | 20.35±7.39 | | 19.68±6.79 | 22.97±3.17 | 29.53±12.99 |  | |  |
|  | TA Type 2x Fiber Percentage (%) | 50.97±8.23 | 46.61±9.32 | 40.41±3.01 | 38.19±16.00 |  | 53.14±6.65 | | 40.72±5.53 | 34.24±5.25^a^ | 47.70±7.72 | # | |  |
|  | TA Type 2b Fiber Percentage (%) | 29.01±7.72 | 27.19±8.18 | 38.8±3.45 | 47.72±15.33^ab^ |  | 24.31±10.21 | | 39.01±9.72 | 43.04±3.73^a^ | 31.14±8.87 | #& | |  |
|  | SS Type 1 Fiber Percentage (%) | 0.20±0.45 | 0.00±0.00 | 0.00±0.00 | 0.00±0.00 |  | 0.00±0.00 | | 0.00±0.00 | 0.20±0.47 | 0.00±0.00 |  | |  |
|  | SS Type 2a Fiber Percentage (%) | 41.59±4.91 | 33.24±5.97 | 34.37±8.44 | 48.79±7.88 |  | 39.24±6.63 | | 37.91±13.36 | 36.71±13.03 | 34.58±8.39 |  | |  |
|  | SS Type 2x Fiber Percentage (%) | 33.92±3.53 | 37.52±8.21 | 29.78±10.02 | 27.82±8.53 |  | 29.85±4.48 | | 22.44±5.34 | 24.63±7.65 | 30.82±4.81 | † | |  |
|  | SS Type 2b Fiber Percentage (%) | 24.39±8.53 | 29.23±6.39 | 35.74±6.94 | 23.39±4.02 |  | 31.03±5.19 | | 41.77±13.09 | 38.27±13.70 | 35.01±8.6 | † | |  |
|  | IS Type 1 Fiber Percentage (%) | 0.00±0.00 | 0.00±0.00 | 0.12±0.27 | 0.00±0.00 |  | 1.63±1.81 | | 0.28±0.26 | 0.00±0.00 | 1.27±1.74 | † | |  |
|  | IS Type 2a Fiber Percentage (%) | 20.39±6.86 | 17.23±5.71 | 29.33±12.60 | 23.98±7.05 |  | 19.43±7.31 | | 20.15±3.69 | 19.54±6.96 | 18.74±15.90 |  | |  |
|  | IS Type 2x Fiber Percentage (%) | 29.63±6.13 | 22.28±3.55 | 22.41±2.63 | 27.92±11.98 |  | 25.05±11.58 | | 20.18±5.38 | 32.60±14.90 | 38.38±15.56 |  | |  |
|  | IS Type 2b Fiber Percentage (%) | 49.98±9.70 | 60.49±7.03 | 48.26±13.58 | 48.09±9.66 |  | 50.69±6.84 | | 59.59±5.67 | 48.48±14.52 | 53.01±27.29 |  | |  |

**Supplemental Table 2: Additional Fiber Data**. Changes in fiber areas by fiber types and fiber type distributions. Data are presented as mean ± standard deviation. Data in rows were analyzed by 2-way ANOVA with significance set at p<0.05. (#) main effect of age, (†) main effect of sex, (&) age-sex interaction, (a) different from 3 MO, (b) different from 9 MO, (c) different from 18 MO.

|  | MALE | | | |  | FEMALE | | | |  | |
| --- | --- | --- | --- | --- | --- | --- | --- | --- | --- | --- | --- |
|  | 9 MO TA | 24 MO TA | 9 MO SS | 24 MO SS |  | 9 MO TA | 24 MO TA | 9 MO SS | 24 MO SS | |  |
| Ifng | 1 | 0.18±0.08^a^ | 0.91±0.28 | 0.16±0.06^a^ |  | 0.56±0.34 | 0.11±0.04 | 0.44±0.36 | 0.14±0.05 | | #†& |
| Tnf | 1 | 4.32±3.23 | 0.41±0.21 | 3.10±0.78 |  | 0.39±0.16 | 5.74±1.87^a^ | 0.40±0.17 | 7.96±3.44^a^ | | #& |
| Il6 | 1 | 2.02±0.82 | 0.62±0.11 | 2.42±1.20 |  | 0.42±0.18 | 2.4±1.47 | 0.74±0.22 | 4.64±3.79^a^ | | # |
| Anxa2 | 1 | 2.50±1.51 | 1.67±1.30 | 3.84±1.59 |  | 0.52±0.21 | 4.92±3.36^a^ | 1.83±0.85 | 7.78±2.54^a^ | | #†& |
| Il4 | 1 | 3.25±1.91 | 1.33±0.29 | 2.19±1.00 |  | 0.77±0.48 | 2.53±1.57 | 1.38±0.61 | 3.68±2.43^a^ | | # |
| Il13 | 1 | 0.61±0.20 | 0.92±0.64 | 0.57±0.28 |  | 0.47±0.37 | 0.70±0.50 | 0.44±0.33 | 1.08±0.61 | | & |
| Myf5 | 1 | 1.33±0.57 | 1.06±0.42 | 1.58±0.68 |  | 0.27±0.28 | 1.51±0.37 | 0.52±0.49 | 2.79±1.50^a^ | | #& |
| Myod1 | 1 | 0.45±0.09 | 1.20±0.48 | 0.62±0.08 |  | 0.56±0.20 | 0.56±0.38 | 0.60±0.12 | 0.60±0.55 | | #& |
| Fbxo32 | 1 | 0.77±0.23 | 1.46±0.43 | 0.74±0.23^a^ |  | 0.71±0.28 | 0.59±0.12 | 0.73±0.10 | 1.05±0.12 | | #†& |
| Trim63 | 1 | 1.21±0.33 | 0.93±0.25 | 1.23±0.26 |  | 0.49±0.16 | 0.98±0.14 | 0.48±0.03 | 1.84±0.38^a^ | | #& |

**Supplemental Table 3: Additional Gene Expression Data**. Changes in expression of the indicated genes normalized to the mean of MALE 9 MO TA. Data are presented as mean ± standard deviation. Data in rows were analyzed by 3-way ANOVA with significance set at p<0.05. (#) main effect of age, (†) main effect of sex, (&) age-sex interaction, (a) different from matched 9 MO.


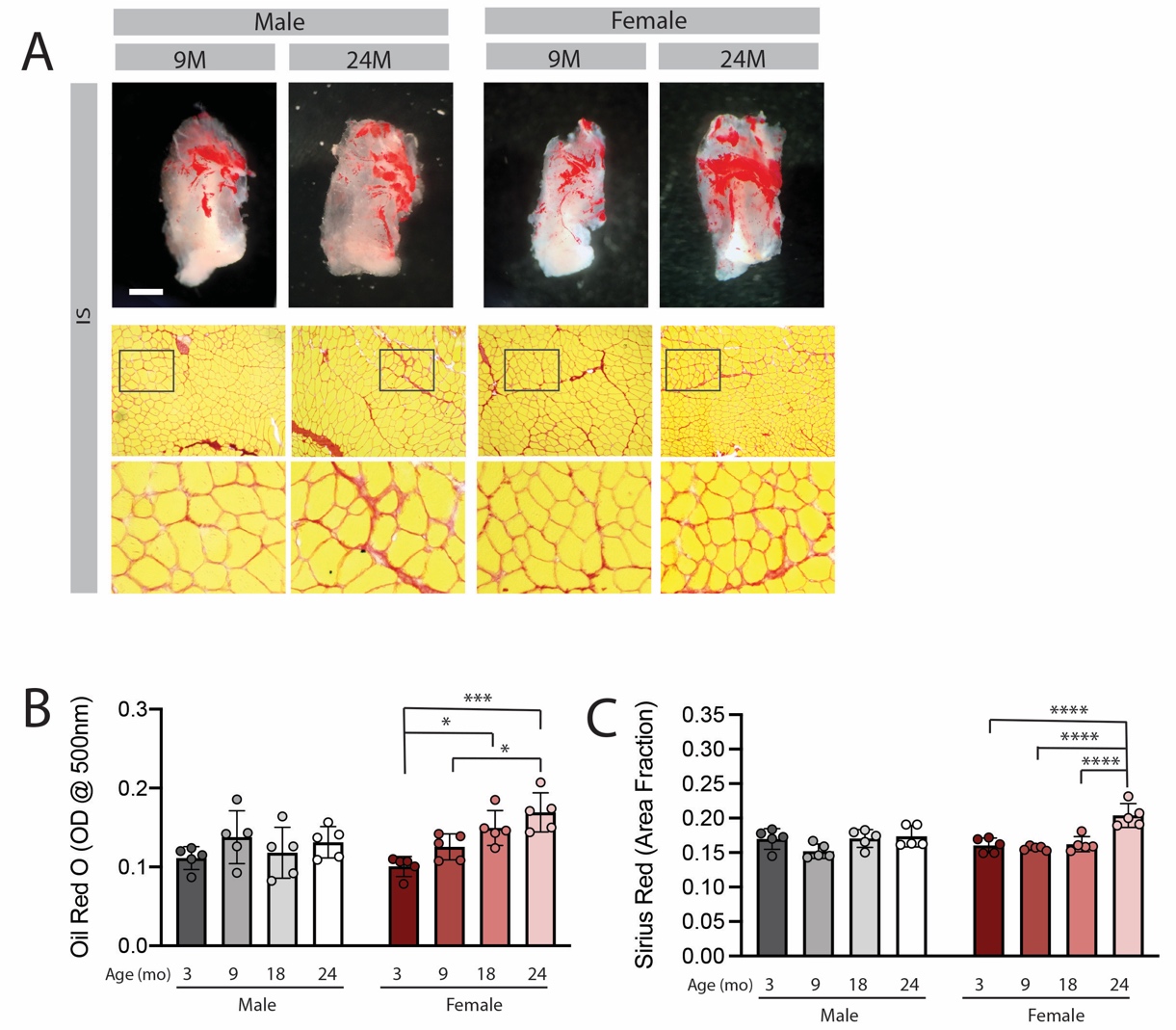


**Fig S1 - The infraspinatus muscle of female mice exhibits fatty infiltration and fibrosis with age.** Representative images of Oil Red O (ORO) stained decellularized muscles (A; top row) and Sirius Red stained histological sections (A; bottom rows). Quantification of extracted ORO shows an increase in intramuscular adipose in the female infraspinatus at 24 months (B). Quantification of the Sirius Red stained area fraction shows an increase in matrix at 24 months (C). 2-way ANOVA of B and C finds a significant main effect of age, but only a significant age-sex interaction for Sirius Red area fraction. * p<0.05, ***p<0.005, ****P<0.001.
