# Supplementary material for: A unique sarcopenic progression in the mouse rotator cuff
Source: J Cachexia Sarcopenia Muscle. 2021 Oct 28;13(1):561–73. doi: 10.1002/jcsm.12808 (PMC8818692; doi:10.1002/jcsm.12808)
Supplement: Supplementary file 2 — Data S1. Supporting information [file JCSM-13-561-s001.docx]

**A Unique Sarcopenic Progression in the Mouse Rotator Cuff**

Gretchen A. Meyer and Karen Shen

*Washington University School of Medicine, St. Louis, MO*

meyerg@wustl.edu

***Contractile Testing***

SS muscle contractile function was assessed as previously described [1]. For this study, this procedure was adapted to assess the contractile function of the IS and TA as well. Briefly, the bony proximal insertion of each muscle was left intact (scapula for the SS and IS, tibia for the TA), but cleared of extraneous musculature. 5-0 braided silk suture was threaded through the bone with a curved needle, knotted and secured to the arm of a dual mode ergometer (305C-LR, Aurora Scientific, Aurora, ON, Canada). The insertion of the distal tendon was left intact and the bone was severed distal to the insertion to leave a bony anchor. The muscle was then transferred and secured in a bath of Ringer’s solution maintained at 37°C and stimulated to contract by parallel plate electrodes connected to an electrical stimulator (701C, Aurora Scientific, Aurora, ON, Canada). Muscle length was increased incrementally until twitch forces plateaued and then adjusted in smaller increments until tetanic force (300 ms train of 0.3 ms pulses at 225 Hz) plateaued. Then, a twitch and tetanic contraction were recorded at this optimal length to assess peak twitch and tetanic tensions and twitch kinetics (time to peak tension and half-relaxation time). Muscle length was determined using a flexible ruler before the muscle was dissected free and weighed. Fiber length and pennation angle were estimated using this measurement, reported values for the muscle/fiber length ratio and pennation angle for each muscle at neutral joint position [2, 3] and a simple geometrical model to account for stretch from the neutral position. For all analyses, force was normalized to physiological cross-sectional area calculated from the above measurements and the density of muscle (1.056 g/cm^3^).

***Quantification of fatty infiltration***

Following contractile testing, IMAT content was quantified as previously described [4]. Briefly, muscles were decellularized in 1% solution of sodium dodecyl sulfate (SDS) which was replaced daily until the muscle was fully transparent (typically 3 days). Decellularized muscles were then fixed in 3.7% formaldehyde overnight and stained with Oil Red O (ORO). These muscles were then cleared in 1% SDS overnight to remove unbound ORO and imaged through a dissecting microscope. Muscles were then placed in 200 μL of isopropanol to extract the stained lipid, and the optical density of the resulting solution was read in duplicate at 500 nm in 75 μL samples in a 96-well plate by Synergy II multi-mode plate reader (Bio-Tek, Winooski, VT).

***Histological Analyses***

Muscles dissected for histological analysis were flash frozen in isopentane cooled by liquid nitrogen. All muscles were cut at the mid-belly into 10 μm sections on a cryostat (Leica Biosystems, Wetzlar, Germany) at −20 °C. Fresh sections were immunostained for myosin heavy chain isoforms (BA-F8, SC-71, and 6H1; Developmental Studies Hybridoma Bank, Iowa City, IA) to determine fiber type distribution and laminin (ab11575; Abcam, Cambridge, UK) to quantify fiber areas. Additional sections were stained with picrosirius red to quantify collagen area fraction and determine fiber numbers. Fiber typing and fiber area analyses were performed using a semi-automated algorithm as previously described [1]. Four non overlapping 20x images from each muscle were analyzed, comprising ~25% of the section; two images were taken in the superficial region and two images were taken in the deep region of the muscle section. Sirius red staining and determination of collagen area fraction were performed as previously described [5]. Collagen area fraction was calculated from two 10x images comprising at least 75% of the section. Muscle fiber number was determined on stitched 4x images comprising the entire muscle section. Fiber borders were detected by thresholding in ImageJ (Mean), and fibers were counted by running the Watershed and Particle Analyzer functions on the resulting mask.

***Transcriptional Analyses***

A portion of the proximal third of each SS and TA muscle from the 9 and 24 month old groups was saved for gene expression analysis. RNA was purified using a combination Trizol/Chloroform extraction and RNEasy kit with DNAse treatment (Qiagen; Hilden, Germany) as per manufacturer’s instructions. Gene expression profiles of 748 genes were analyzed using the Nanostring "nCounter XT Codeset Gene Expression Assays" protocol by the Genome Technology Access Center (Washington University, St. Louis, MO). Twenty-four samples (n=3 per group) were processed according to manufacturer's recommendations. Hybridization of the RNA to the XT Mouse Metabolic Pathways Codeset was performed with inputs of 100ng of total RNA per sample. Following hybridization, samples were processed on the Nanostring Prep Station where they were purified and immobilized on a sample cartridge for data collection. Images of the immobilized fluorescent reporters in the sample cartridge were taken via the Digital Analyzer where the maximum of 555 Fields of View were collected. Resulting RCC files were imported into the nSolver Analysis Software for QC and analysis. There were no QC flags. Binding densities ranged from 0.89 to 1.81. Advanced Analysis was performed on all samples with a categorical reference of 9 month old male TA. Clustering was performed on pathway Z-scores by pathway and by sample. Cell type profiling was also used to identify cell types expected to have altered representation based on cell type-specific marker genes.

***Quantitative PCR***

Remaining RNA was used to assay transcription of genes of interest that were not included in the Metabolic Pathways panel. Briefly, cDNA was created using superscript III reverse transcriptase (Life Technologies) according to manufacturer instructions. Transcript copies were detected using Fast SYBR Green (Applied Biosystems, Foster City, CA) using primer sequences listed below. Reactions were run in duplicate with the following reaction profile: 2 minutes at 50°C and 10 minutes at 95°C, followed by 40 cycles of 15 seconds at 95°C and 1 minute at 60°C. All expression values were normalized to expression of GAPDH.

| Gene | Forward Primer | Reverse Primer |
| --- | --- | --- |
| Pparg | GCATGGTGCCTTCGCTGA | TGGCATCTCTGTGTCAACCATG |
| Adipoq | GCACTGGCAAGTTCTACTGCAA | GTAGGTGAAGAGAACGGCCTTGT |
| Tgfb1 | TGCGCTTGCAGAGATTAAAA | CGTCAAAAGACAGCCACTCA |
| Col1a1 | GCTCCTCTTAGGGGCCACT | CCACGTCTCACCATTGGGG |
| Ifng | ACCACAGTCCATGCCATCAC | TCCACCACCCTGTTGCTGGTA |
| Tnf | CCCCAAAGGGATGAGAAGTT | CACTTGGTGGTTTGCTACGA |
| Il6 | GAACAACGATGATGCACTTGC | CTTCATGTACTCCAGGTAGCTATGGT |
| Anxa2 | TCAGACAAATACTTCCATGCT | AAAGGCCTCTCTTCCATCACT |
| Il4 | GAATAGGCCGGTCCAATCAGA | CAGCCATTCGTCGGACACATT |
| Il13 | CAGCCTCCCCGATACCAAAAT | GCGAAACAGTTGCTTTGTGTAG |
| Pdgfra | AGGTCCAGGGAGGTTGTGA | CCGCCATGTAGTCCAGGTAG |
| Fbxo32 | AACCGGGAGGCCAGCTAAAGAACA | TGGGCCTACAGAACAGACAGTGC |
| Trim63 | GAGAACCTGGAGAAGCAGCT | CCGCGGTTGGTCCAGTAG |
| Myf5 | TGAGGGAACAGGTGGAGAAC | TGGAGAGAGGGAAGCTGTGT |
| Myod1 | CCTAAGCGACACAGAACAGG | CTTTTGGGCGTGAAGAACC |

***Inflammatory Signaling Network***

A signaling network including infiltrating immune cells, fibro/adipogenic progenitors (FAPs), atrophic fibers and activated satellite cells was constructed based on published muscle-specific pathways [6]. This network was updated with recently published data on the interaction of tumor necrosis factor α (TNFα) [7], transforming growth factor β (TGFβ) [8], interleukin 6 (IL-6) [8], annexin A2 [9], interleukin 4 (IL-4) [10] and interleukin 13 (IL-13) [10] with FAPs. Gene nodes were colored according to the expression level in 24 month muscles compared with 9 month samples in the indicated muscles. Gray nodes represent genes for which transcriptional data do not exist.

***Flow Cytometry***

Quantification of fibroadipogenic progenitors was performed via flow cytometry as previously described [11]. Briefly, the tibialis anterior and supraspinatus muscles were minced and incubated in a digestive solution containing 2.67 mg/mL Collagenase type I, 75 mg/mL dispase II and 1% penicillin/streptomycin in high glucose DMEM. Tissue was mechanically dissociated during digestion and then cells were filtered, centrifuged and resuspended in blocking buffer containing 1mM EDTA and 2.5% goat serum in PBS. Primary antibodies were added to the buffer as follows: CD45-eFluor 450 (48-0451-82; Invitrogen; 1:200), CD31-eFluor 450 (48-0311-82; Invitrogen; 1:200), Ly-6A/E (Sca-1)-FITC (553335; BD Pharmigen; 1:200). Following 20 minutes of incubation on ice, samples were centrifuged and resuspended in blocking buffer for analysis on an LSR Fortessa (BD) flow cytometer. Fibroadipogenic progenitors were identified as the percentage of CD45-/CD31- single cells positive for Sca-1.

References:

1. Bryniarski AR, Meyer GA. Brown Fat Promotes Muscle Growth During Regeneration. J Orthop Res. 2019;37:1817-26. doi:10.1002/jor.24324

2. Mathewson MA, Kwan A, Eng CM, Lieber RL, Ward SR. Comparison of rotator cuff muscle architecture between humans and other selected vertebrate species. J Exp Biol. 2014;217:261-73. doi:10.1242/jeb.083923

3. Burkholder TJ, Fingado B, Baron S, Lieber RL. Relationship between muscle fiber types and sizes and muscle architectural properties in the mouse hindlimb. J Morphol. 1994;221:177-90. doi:10.1002/jmor.1052210207

4. Biltz NK, Meyer GA. A novel method for the quantification of fatty infiltration in skeletal muscle. Skelet Muscle. 2017;7:1. doi:10.1186/s13395-016-0118-2

5. Meyer GA. Evidence of induced muscle regeneration persists for years in the mouse. Muscle Nerve. 2018;58:858-62. doi:10.1002/mus.26329

6. Smith LR, Meyer G, Lieber RL. Systems analysis of biological networks in skeletal muscle function. Wiley Interdiscip Rev Syst Biol Med. 2013;5:55-71. doi:10.1002/wsbm.1197

7. Lemos DR, Babaeijandaghi F, Low M, Chang CK, Lee ST, Fiore D, et al. Nilotinib reduces muscle fibrosis in chronic muscle injury by promoting TNF-mediated apoptosis of fibro/adipogenic progenitors. Nat Med. 2015;21:786-94. doi:10.1038/nm.3869

8. Madaro L, Passafaro M, Sala D, Etxaniz U, Lugarini F, Proietti D, et al. Denervation-activated STAT3-IL-6 signalling in fibro-adipogenic progenitors promotes myofibres atrophy and fibrosis. Nat Cell Biol. 2018;20:917-27. doi:10.1038/s41556-018-0151-y

9. Hogarth MW, Defour A, Lazarski C, Gallardo E, Diaz Manera J, Partridge TA, et al. Fibroadipogenic progenitors are responsible for muscle loss in limb girdle muscular dystrophy 2B. Nat Commun. 2019;10:2430. doi:10.1038/s41467-019-10438-z

10. Heredia JE, Mukundan L, Chen FM, Mueller AA, Deo RC, Locksley RM, et al. Type 2 innate signals stimulate fibro/adipogenic progenitors to facilitate muscle regeneration. Cell. 2013;153:376-88. doi:10.1016/j.cell.2013.02.053

11. Meyer GA, Lieber RL. Skeletal muscle fibrosis develops in response to desmin deletion. Am J Physiol Cell Physiol. 2012;302:C1609-20. doi:10.1152/ajpcell.00441.2011
